# Mycobacterial Infections in Patients With Hairy Cell Leukemia: A Systematic Review of Published Cases

**DOI:** 10.1093/ofid/ofag063

**Published:** 2026-02-11

**Authors:** Praveen Kumar Tirlangi, Venkata Swathi Kiran Pothumarthy, Adil Rashid Khan, Santosh Kumar Chellapuram, Martin Peter Grobusch, Nitin Gupta

**Affiliations:** Department of Infectious Diseases, Kasturba Medical College, Manipal Academy of Higher Education, Manipal, Karnataka, India; Department of Infectious Diseases, Kasturba Medical College, Manipal Academy of Higher Education, Manipal, Karnataka, India; Department of Medicine, All India Institute of Medical Sciences, Ansari Nagar, New Delhi, India; Medical Oncology & BMT Unit, Star Hospitals, Hyderabad, Telengana, India; Center of Tropical Medicine and Travel Medicine, Department of Infectious Diseases, Amsterdam University Medical Centers, University of Amsterdam, Amsterdam Public Health—Global Health, Amsterdam Infection & Immunity, Amsterdam, The Netherlands; Masanga Medical Research Unit (MMRU), Masanga, Sierra Leone; Centre de Recherches Médicales en Lambaréné, Gabon; Institut für Tropenmedizin und Deutsches Zentrum für Infektiologie (DZIF), Universität Tübingen, Tübingen, Germany; Institute of Molecular Medicine and Infectious Diseases, University of Cape Town, Cape Town, South Africa; Department of Infectious Diseases, Kasturba Medical College, Manipal Academy of Higher Education, Manipal, Karnataka, India

## Abstract

**Background:**

Hairy cell leukemia (HCL) is a rare B-cell lymphoproliferative disorder characterized by monocytopenia and profound defects in cellular immunity, predisposing patients to severe mycobacterial infections. Although numerous case reports exist, the clinical features and outcomes of these infections have not been systematically synthesized.

**Methods:**

We conducted a systematic review following PRISMA guidelines, searching PubMed, Embase, and Web of Science from inception to 31 January 2025, without language restrictions. Studies were eligible if they contained individual patient-level data on microbiologically confirmed mycobacterial infection in HCL. Data on demographics, clinical presentation, organ involvement, pathogen distribution, treatment timing, and outcomes were extracted and analyzed descriptively.

**Results:**

Thirty-six articles describing 48 patients met the inclusion criteria. The mean age was 53.7 ± 12.7 years, and 39/48 (81%) were male. Disseminated infection was common, occurring in 34/48 (71%) cases, with frequent involvement of lymph nodes (60%), lungs (56%), liver (27%), spleen (25%), and bone marrow (19%). Infections were caused by *Mycobacterium tuberculosis* complex in 12/48 (25%) and by nontuberculous mycobacteria in 33/48 (69%), most commonly *M. kansasii* (55%) and *M. avium* complex (30%). Overall, 19/48 (40%) patients died, with significantly higher mortality among those with pulmonary involvement (79% vs 41%; *P* = .01).

**Conclusions:**

Mycobacterial infections in HCL are frequently disseminated and associated with substantial mortality. Clinicians should maintain a high index of suspicion, ensure timely species-level diagnosis, and evaluate for multisystem involvement when managing suspected mycobacterial disease in HCL.

## Teaser

This systematic review synthesizes published cases of mycobacterial infections in hairy cell leukemia, demonstrating frequent dissemination, predominance of nontuberculous mycobacteria, and substantial mortality. Early clinical suspicion, timely species-level diagnosis, and comprehensive evaluation are critical to improving outcomes in this highly immunocompromised population.

Hairy cell leukemia (HCL) is a rare indolent B-cell neoplasm characterized by pancytopenia, splenomegaly, bone marrow fibrosis, and lymphocytes with distinctive cytoplasmic “hairy” projections [1–3]. Men and individuals of white ethnicity have a higher incidence, most common in middle-aged-to-older adults [1–3]. The neoplastic lymphocytes display aberrant expression of surface proteins that influence their tissue localization and interactions with the microenvironment. CD11c, an adhesion molecule typically found on monocytes and macrophages, mediates binding to complement-coated microbes [4–6]. In HCL, although the leukemic B cells express CD11c, they lack the antimicrobial effector functions of professional phagocytes, allowing intracellular pathogens such as *Mycobacterium* spp. to persist [7]. CD103 and CXCR4 further determine tissue homing and retention. CD103 binds E-cadherin, anchoring cells within the bone marrow and splenic red pulp, whereas CXCR4 guides migration toward these same niches in response to CXCL12. Once localized, adhesion is reinforced through VCAM-1 interactions, which are upregulated by TNF-α during the inflammatory process. These mechanisms promote the accumulation of leukemic cells in the marrow, liver, and spleen, while the absence of CXCR5 and CCR7 prevents lymph node homing [8, 9]. This unique trafficking pattern, coupled with impaired antimicrobial function, enables HCL cells to serve as permissive reservoirs for intracellular pathogens such as *Mycobacterium* spp. In addition, HCL creates a profoundly immunosuppressed milieu through monocytopenia, lymphopenia, NK-cell dysfunction, marrow infiltration, hypersplenism, and elevated TNF-α, resulting in pancytopenia and markedly reduced antimicrobial defenses [7, 10–12]. Severe fungal (*Cryptococcus* spp., *Histoplasma* spp.) and bacterial (*Pseudomonas* spp., *Escherichia coli*) infections occur in up to 40% of patients [11]. Mycobacterial infections are a particular concern, especially in endemic regions, where they may manifest as pulmonary, extrapulmonary, or disseminated disease. Notably, infection can occasionally precede the diagnosis of HCL, underscoring the interplay between disease biology and infection risk [12–14]. To better define this association, we conducted a systematic review of individual patient data to characterize the spectrum, timing, and outcomes of mycobacterial infections in HCL, and to explore their relationship with disease biology and therapy.

## METHODS

### Study Design and Objective

This systematic review was conducted after registering in the PROSPERO database (CRD420250651278) in accordance with the Preferred Reporting Items for Systematic Reviews and Meta-Analyses (PRISMA) guidelines (PRISMA checklist attached as supplementary file) [15]. The primary objective was to examine the relationship between HCL and mycobacterial infections, with a focus on clinical presentation, organ involvement, diagnostic methods, treatment strategies, and outcomes. Only reports with individual patient-level data were included to allow for detailed and granular analysis.

### Search Strategy

A comprehensive search was conducted across PubMed, Embase, and Web of Science, spanning the databases’ inception to 31 January 2025. The following search string was used: (“hairy cell”) AND (leukaem* OR leukem*) AND (infect* OR TB OR tubercul* OR mycobact*). The reference lists of relevant articles were also manually screened to ensure completeness. No language restrictions were applied.

### Eligibility Criteria

Studies were eligible if they were published case reports or case series describing patients with a confirmed diagnosis of HCL and providing individual-level data on concomitant or subsequent mycobacterial infection. Reports were required to include details on presentation, diagnosis, treatment, or outcomes. Review articles, editorials, conference abstracts, and studies without individual patient details were excluded, as were studies lacking microbiological confirmation of mycobacterial infection.

### Study Selection and Data Extraction

Two reviewers (P. K. T. and V. S. K. P.) independently screened titles, abstracts, and full texts. Any disagreements were resolved through discussion or by consultation with a third reviewer (N. G.). A standardized data extraction form was developed a priori and used across all studies. Extracted data (entered by 1 reviewer and verified by another) included demographic details, hematological parameters at the time of diagnosis of mycobacterial infection, timing of mycobacterial infection in relation to HCL diagnosis and treatment, type of mycobacteria and diagnostic methods, clinical features and organ involvement, HCL-directed therapies, treatment of mycobacterial infection, and outcomes including remission, relapse, and mortality.

### Definitions

Microbiological confirmation of mycobacterial infection was defined as culture or molecular test positivity from any clinical specimen. Organ involvement was attributed when microbiological confirmation was obtained from tissue or aspirate of the affected site. For organs where primary HCL involvement is uncommon, such as the lungs, lymph nodes, or central nervous system (CNS), involvement was inferred if compatible clinical or radiological findings were present alongside microbiological confirmation from another site. Disseminated mycobacterial infection was defined a priori as involvement of 2 or more noncontiguous organ systems (eg, lung, liver, spleen, lymph nodes, bone marrow, skin, and central nervous system), and/or the isolation of *Mycobacterium* spp. from a sterile site, including blood, bone marrow, liver, or spleen. This definition aligns with established criteria used in immunocompromised hosts and was applied uniformly to all cases. The temporal relationship between mycobacterial infections and HCL treatment was classified as either occurring before or after initiation of HCL-directed therapy. Monocytopenia was defined as an absolute monocyte count <200 cells/µL (0.2 × 10⁹/L), and lymphopenia as an absolute lymphocyte count <1000 cells/µL (1.0 × 10⁹/L) in adults. In instances where individual reports did not specify absolute counts but explicitly described the patient as having monocytopenia or lymphopenia, such cases were also recorded as fulfilling the respective definition.

### Risk of Bias Assessment

All included case reports were critically appraised using the Joanna Briggs Institute (JBI) Critical Appraisal Checklist for Case Reports [16]. Each report was evaluated across key domains, demography, patient history, clinical presentation, diagnostic work-up, treatment, and follow-up, with items judged as adequately/comprehensively reported, inadequately reported, or absent. Patient history was considered adequate only if it included both relevant clinical details and lymphocyte or monocyte counts at the time of mycobacterial diagnosis. Adverse events, although part of the original JBI tool, were considered irrelevant to the objectives of this review and were not evaluated. Two reviewers (P. K. P. and V. S. K. P.) independently conducted the appraisal, and disagreements were resolved through discussion.

### Data Analysis

Descriptive statistics were used to summarize patient characteristics, clinical features, organ involvement, treatment modalities, and outcomes. Categorical variables were expressed as absolute numbers and percentages, while continuous variables were reported as means with standard deviations. For comparative analysis, patients were stratified according to the type of mycobacterial infection: *Mycobacterium tuberculosis* complex (MTBC), *Mycobacterium avium* complex (MAC), and *M. kansasii*. Several stratified analyses were conducted to explore patterns of dissemination and organ involvement. Differences in categorical variables between groups were assessed using the χ^2^ test or Fisher's exact test, as appropriate. A *P* value of <.05 was considered statistically significant.

## RESULTS

### Study Selection

**Figure 1.**
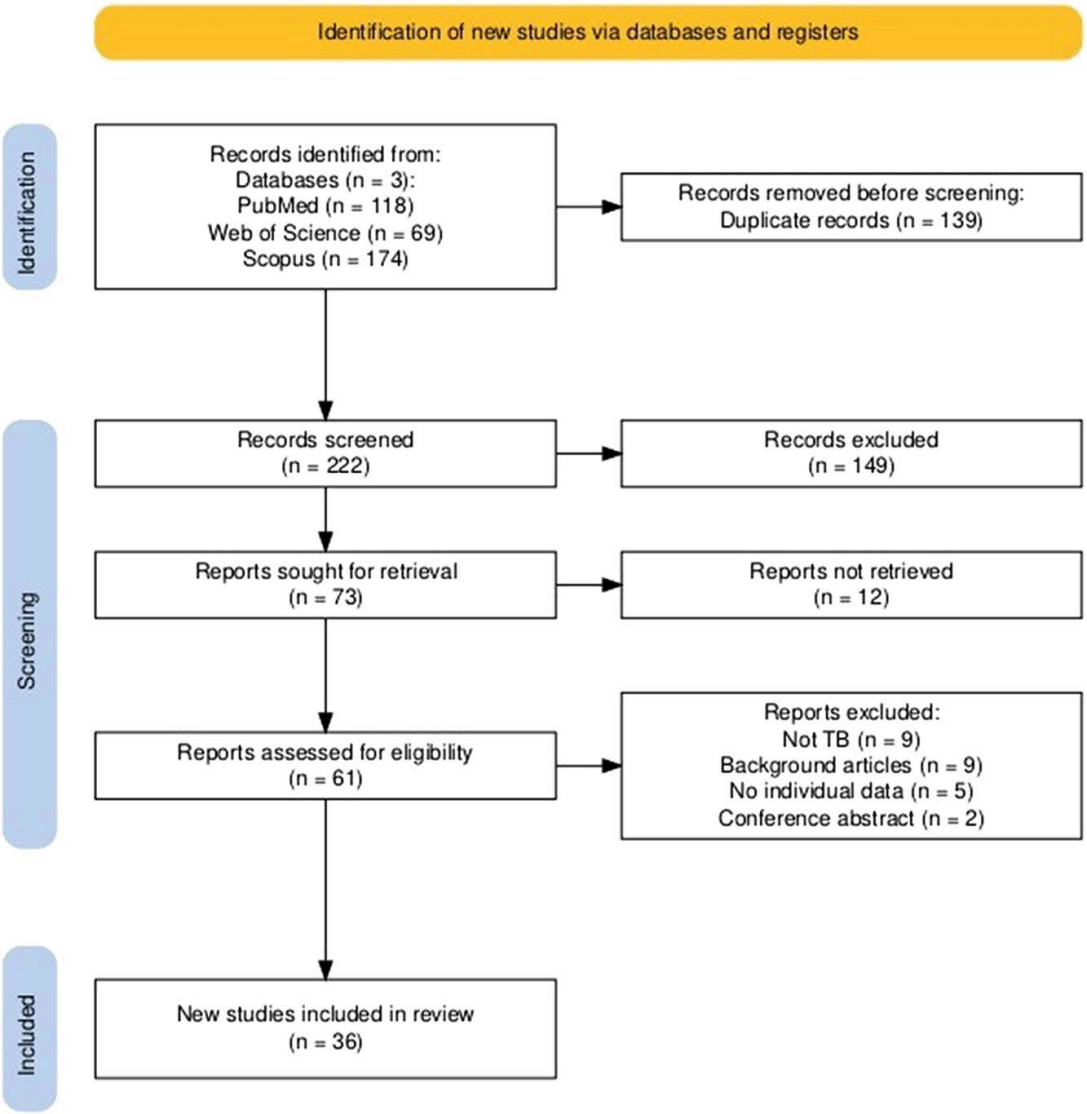
PRISMA flow diagram depicting the selection process for studies included in the systematic review.

The initial database search yielded 361 records (PubMed: 118, Web of Science: 69, Scopus: 174). After removing 139 duplicates, 222 records remained for screening. Title and abstract screening excluded 149 records (67%), leaving 73 articles for full-text review. Of these, 37 were excluded (full text unavailable, n = 12; 32.4%, no microbiologically proven mycobacterial infection, n = 9; 24.3%, background articles, n = 9; 24.3%, no individual patient data, n = 5; 13.5%, conference abstracts, n = 2; 5.4%). Ultimately, 36 articles reporting 48 individual cases were included [13, 17–50] (Figure 1).

### Critical Appraisal of Included Studies

Of the 48 individual cases appraised, demographic details, treatment, and follow-up were consistently reported (100%) [13, 17–50] (Supplementary Table 1). Clinical presentation was adequately described in 47 reports (97.9%), and diagnostic methods were appropriately detailed in 44 (91.7%). Patient history was less comprehensive, with only 17 reports (35.4%) providing sufficient detail. Adverse events were not evaluated for this review. Overall, reporting quality was high, with the main limitation being incomplete patient histories.

### Study and Patient Characteristics

The included studies were published between 1976 and 2024 [13, 17–50]. Over half (19/36, 53%) were published before 2000 [13, 17–19, 22–25, 33–42, 48, 49]. Geographically, most originated from the United States (11/36, 30.5%) and the United Kingdom (5/36, 14%) [13, 17, 22, 23, 26–29, 33–38, 40, 41]. Among the 48 cases, 81% (n = 39) were male (Table 1). The mean age at presentation was 53.7 ± 12.7 years. In all cases, mycobacterial infections were diagnosed after HCL. Fever was reported in 90% (n = 43). The median absolute lymphocyte and monocyte count at the time of diagnosis of mycobacterial infections was 10 (0–32)/mcL and 850 (260–1232)/mcL, respectively. Monocytopenia and lymphopenia were present in 95.6% (22/23) and 68.2% (15/22) patients, respectively. In 63% (30/48) of the patients, mycobacterial infections were identified before initiation of HCL-directed therapy. Reported HCL treatments included steroids (14/48, 29%), interferon-α (8/48, 17%), cladribine (12/48, 25%), rituximab (6/48, 13%), chlorambucil (5/48, 10%), and pentostatin (2/48, 4%). Splenectomy was performed in 46% (22/48) of cases (Table 1).

**Table 1.** Reported Cases of Mycobacterial Infections in Patients With Hairy Cell Leukemia (HCL) Included in the Systematic Review

An	Author	Year	Country	Age	Sex	Mycobacteria spp. Diagnosis In Relation To HCL	Dissemination	Etiology	Mortality
1	Cellini [18]	2022	Italy	59	Male	Before treatment	Yes	Mtb	No
2	Castor [19]	1994	Sweden	57	Female	Before treatment	Yes	*M. malmoense*	Yes
3	Broady [20]	1999	Australia	57	Male	Before treatment	Yes	MAC	No
4	Haefliger [21]	2023	Switzerland	74	Male	Before treatment	No	MAC	Yes
5	Dugdale [22]	1989	USA	58	Male	Before treatment	Yes	MAC	No
6	Bain [23]	1992	UK	67	Male	Before treatment	No	Mtb	No
7	De Kruijf [24]	1997	Netherlands	72	Female	Before treatment	Yes	*M. bovis*	Yes
8	Hulin [25]	1990	France	77	Male	Before treatment	No	Mtb	No
9	Trizna [26]	2001	USA	60	Female	After treatment	No	Mk	No
10	Fleisher	2006	Brazil	44	Female	Before treatment	Yes	*M*. *abscessus*	No
11	Geller [27]	2015	USA	67	Male	After treatment	Yes	Mtb	No
12	Thaker [28]	2001	UK	51	Male	After treatment	Yes	MAC	No
				44	Female	Before treatment	No	Mtb	No
13	Green [29]	2008	UK	70	Male	After treatment	Yes	Mk	Yes
14	Valizadeh [30]	2012	Iran	46	Male	After treatment	Yes	Mtb	No
15	Papadopoulos [31]	2010	Greece	69	Male	After treatment	Yes	AFB	No
16	Gogia [32]	2013	India	52	Male	After treatment	No	Mtb	No
17	Weinstein [33]	1978	USA	28	Male	Before treatment	Yes	Mk	Yes
				57	Male	Before treatment	Yes	MAC	No
18	Manes [34]	1976	USA	75	Male	Before treatment	Yes	Mk	Yes
19	Raanani [35]	1996	Israel	62	Female	After treatment	No	Mk	No
20	Dave [36]	1993	UK	55	Male	Before treatment	Yes	*M. malmoense*	No
21	Bennett [13]	1986	USA	65	Male	Before treatment	Yes	Mtb	Yes
				30	Male	After treatment	No	Mk	Yes
				60	Male	Before treatment	No	Mk	No
				28	Male	Before treatment	Yes	Mk	Yes
				46	Female	Before treatment	Yes	Mk	No
				33	Male	After treatment	Yes	*M*. *chelonae*	Yes
				57	Male	Before treatment	Yes	Mk	No
				49	Male	After treatment	No	MAC	No
				42	Male	Before treatment	No	MAC	No
22	Rice [37]	1982	USA	54	Male	After treatment	Yes	Mk	No
				50	Male	Before treatment	Yes	Mk	Yes
23	Weinstein [38]	1981	USA	59	Male	After treatment	Yes	Mk	Yes
				50	Female	After treatment	Yes	Mk	No
24	Mead [39]	1983	UK	48	Female	Before treatment	Yes	Mk	Yes
25	Maziarz [40]	1988	USA	47	Male	Before treatment	Yes	MAC	No
26	Hendrick [41]	1979	USA	55	Male	Before treatment	Yes	*M*. *scrofulaceum*	Yes
27	Maurice [42]	1988	Australia	66	Male	After treatment	Yes	MAC	No
28	Mummler [43]	2021	Germany	62	Male	Before treatment	Yes	Mk	No
29	Fonseca [44]	2016	Portugal	67	Male	After treatment	Yes	Mtb	No
30	Ramasamy [45]	2014	India	50	Male	After treatment	No	AFB	Yes
31	Girardi [46]	2012	Italy	57	Male	After treatment	No	Mtb	Yes
32	Filho [47]	2011	Brazil	35	Male	Before treatment	Yes	AFB	No
33	Kramers [48]	1992	Netherlands	40	Male	Before treatment	Yes	Mk	No
34	Nielsen [49]	1981	Denmark	39	Male	Before treatment	No	MAC	Yes
35	Arslan [50]	2013	Turkey	56	Male	Before treatment	Yes	Mtb	Yes
36	Stanton [17]	2024	USA	31	Male	Before treatment	Yes	Mk	Yes

Abbreviations: HCL, hairy cell leukemia; Mtb, *Mycobacterium tuberculosis*; Mk, *Mycobacterium kansasii*; MAC, *Mycobacterium avium* complex; AFB, acid-fast bacilli (not further speciated); *M. scrofulaceum*, *Mycobacterium scrofulaceum*; *M. chelonae*, *Mycobacterium chelonae*; *M. abscessus*, *Mycobacterium abscessus*; *M. malmoense*, *Mycobacterium malmoense*; *M. bovis*, *Mycobacterium bovis*.

### Clinical Profile of Mycobacterial Infections

Dissemination was reported in 71% (34/48) of patients (Table 1). The distribution of organ involvement was lymph nodes (29/48, 60%), lungs (27/48, 56%), liver (13/48, 27%), spleen (12/48, 25%), bone marrow (9/48, 19%), skin (7/48, 15%), pleura (6/48, 13%), and CNS (4/48, 8%). Blood cultures were positive for mycobacteria in 36% (12/33) of the tested patients. Granulomas were histopathologically demonstrated in 63% (30/48) of cases.

### Microbiological Spectrum

Mycobacterial infections were attributed to MTBC in 12 cases (25%) and to nontuberculous mycobacteria (NTM) in 33 cases (69%); species distinction was not reported in 3 cases (6%). Among NTMs, the most frequent isolates were *M. kansasii* (18/33, 55%) and MAC (10/33, 30%) (Table 2).

**Table 2.** Comparison of Clinical Features and Outcomes Among Patients With MTBC, MAC, and *M. kansasii*

Feature	MTBC (n = 12)	MAC (n = 10)	*M. kansasii* (n = 18)	*P V*alue
Male gender	11/12 (92%)	8/10 (80%)	16/18 (89%)	.692
Fever	10/12 (83%)	10/10 (100%)	13/18 (72%)	.179
Mycobacterial infection diagnosed before HCL treatment	7/12 (58%)	7/10 (70%)	11/18 (61%)	.842
Dissemination	7/12 (58%)	6/9 (67%)^a^	14/18 (78%)	.518
Lung involvement	6/12 (50%)	4/10 (40%)	11/18 (61%)	.551
CNS involvement	4/12 (33%)	0/10 (0%)	0/18 (0%)	.**006**
Blood culture positivity	4/10 (40%)^a^	3/5 (60%)^a^	3/10 (30%)^a^	.535
Granulomas^b^	6/12 (50%)	7/10 (70%)	13/18 (72%)	.425
Death	4/12 (33%)	2/10 (20%)	9/18 (50%)	.273

Abbreviations: MTBC, *Mycobacterium tuberculosis* complex; MAC, *Mycobacterium avium* complex; HCL, hairy cell leukemia; CNS, central nervous system.

^a^Denominators vary due to incomplete reporting across studies.

^b^Granulomas—identified on histopathological examination.

### Comparative Analysis by Pathogen

Key clinical features and outcomes among patients with MTBC (n = 12), MAC (n = 10), and *M. kansasii* (n = 18) are summarized in Table 2. Central nervous system involvement was observed exclusively in MTBC cases (4/12, 33%; *P* = .006). Mortality was highest with *M. kansasii* (9/18, 50%) compared with MTBC (4/12, 33%) and MAC (2/10, 20%), though not statistically significant (*P* = .273).

### Dissemination of Mycobacterial Infection

Dissemination was a dominant clinical pattern across the cohort, and stratified analyses were undertaken to characterize its timing, extent, and pathogen-specific behavior (Supplementary Tables 2–6). Disseminated infection was frequent in both groups, identified in 76.7% of cases diagnosed before HCL therapy and 61.1% of those diagnosed after therapy (*P* = .251). Similarly, mycobacteremia occurred at similar rates before and after therapy (38.1% vs 33.3%; *P* = .784) (Supplementary Table 2). A comparison of disseminated and nondisseminated infections revealed no significant differences in cytopenias, granulomatous inflammation, or mortality (all *P* > .05), indicating that dissemination reflects underlying host susceptibility rather than clinical severity markers (Supplementary Table 3). Species-level analyses showed that dissemination was common with both MTBC (58.3%) and NTM (75.0%; *P* = .281), with broadly overlapping involvement of lymph nodes, liver, spleen, bone marrow, and lungs (Supplementary Table 4). Restricting the analysis to disseminated cases alone yielded similar patterns of organ involvement between MTBC and NTM (all *P* > .05) (Supplementary Table 5). Pulmonary disease was rarely localized: among 25 pulmonary infections with identifiable species, all MTBC cases (100%) and most NTM cases (84.2%; *P* = .299) had concurrent extrapulmonary involvement (Supplementary Table 6). Together, these findings establish dissemination as the predominant presentation of mycobacterial disease in HCL across treatment groups and pathogen categories.

### Factors Associated With Mortality

Nineteen patients (40%) died during follow-up. Most clinical and laboratory variables did not differ significantly between patients with and without mortality in HCL with concomitant mycobacterial infection (Table 3). Male gender, fever, timing of diagnosis of mycobacterial infection in relation to HCL treatment, monocytopenia, lymphopenia, dissemination, and blood culture positivity for mycobacteria were comparably distributed across both groups. However, pulmonary involvement was significantly more frequent among patients who died (78.9%) compared with those who survived (41.4%, *P* = .01).

**Table 3.** Comparison of Clinical and Laboratory Variables Between Patients With and Without Mortality in Hairy Cell Leukemia With Concomitant Tuberculosis

Variable	Death (n = 19)	No Death (n = 29)	*P V*alue
Male gender	16/19 (84.2%)	23/29 (79.3%)	.671
Fever	18/19 (94.7%)	25/29 (86.2%)	.344
Mycobacterial infection diagnosis before HCL treatment	13/19 (68.4%)	17/29 (58.6%)	.493
Monocytopenia	11/11 (100%)	11/12 (91.7%)	.328
Lymphopenia	5/8 (62.5%)	10/14 (71.4%)	.665
Dissemination	14/19 (73.7%)	20/28 (71.4%)	.865
Lung involvement	15/19 (78.9%)	12/29 (41.4%)	.010
Blood culture positive for Mycobacteria spp.	5/14 (35.7%)	7/19 (36.8%)	.947

Values are presented as proportions (number with variable/total assessed). *P* values are from Chi-square or Fisher's exact test, as appropriate.

Abbreviations: TB, tuberculosis; HCL, hairy cell leukemia.

Cause-specific mortality attribution was not possible due to incomplete reporting in older case reports.

## DISCUSSION

This systematic review synthesizes nearly 5 decades of published reports on mycobacterial infections in HCL. Infections frequently occurred before the initiation of HCL therapy, were predominantly disseminated, and were most often caused by NTM, particularly *M. kansasii* and MAC. Central nervous system involvement was observed only in MTBC cases. Despite therapy, overall mortality remained high (40%), with the highest rates among *M. kansasii* cases (50%). These findings underscore that mycobacterial disease in HCL reflects not only environmental exposure but also the convergence of distinct host immune defects with microbial virulence strategies [13, 17–50].

The burden of mycobacterial infections in HCL varies markedly across geography and time, raising the possibility that some of the observed patterns may reflect regional background epidemiology rather than a disease-specific association. Early retrospective series from the United States in the 1980s reported prevalences ranging from 4.8% to over 20%, predominantly due to atypical mycobacteria such as *M. kansasii* and MAC [12, 13, 38, 51, 52], while Indian cohorts have documented similarly high rates, with 11%–13% of patients developing tuberculosis, including disseminated and multidrug-resistant forms [14, 53]. In contrast, recent large European multicenter cohorts describe mycobacterial infections as rare (<1%) [54, 55]. These geographic discrepancies raise a legitimate alternative explanation: some differences in MTBC versus NTM distribution may simply mirror local pathogen prevalence, and in high-burden regions, tuberculosis may occasionally lead to the incidental discovery of underlying HCL. Publication bias further contributes, as most reported cases originate from high-income nations, whereas under-reporting from TB-endemic regions in Asia, Africa, and Latin America is likely [13, 17, 22, 23, 26–29, 33–38, 40, 41]. However, even when considering these contextual factors, the uniformly high rates of dissemination across diverse settings suggest that environmental exposure alone cannot account for the clinical phenotype. Data on other opportunistic infections in HCL, such as invasive fungal or viral pathogens, remain sparse, limiting the ability to determine whether mycobacterial dissemination is unique or part of a broader pattern of susceptibility. Comparative studies across different pathogens and epidemiologic backgrounds will be critical to disentangling the contributions of exposure from the intrinsic immunologic vulnerabilities characteristic of HCL.

The association between mycobacterial infection and HCL likely reflects the convergence of pathogen biology with the characteristic immune deficits of the disease. Pathogenic mycobacteria rely on specialized secretion systems to persist within host cells, while patients with HCL exhibit monocytopenia, lymphopenia, NK-cell dysfunction, and splenic sequestration, defects that collectively impair intracellular pathogen control [56, 57]. Consistent with this biology, more than 70% of infections in this review were disseminated, with frequent involvement of the lungs, liver, spleen, bone marrow, skin, and lymph nodes, and mycobacteremia documented in over one-third of cases. Granuloma formation remained intact in many patients, suggesting partial preservation of inflammatory containment despite impaired cell-mediated immunity. The consistently high rates of multiorgan involvement across subgroups, including cases with primary pulmonary presentation, further indicate that the immunologic milieu of HCL limits effective containment regardless of the mycobacterial species. That pulmonary MTBC and NTM infections were rarely isolated but instead almost universally accompanied by extrapulmonary involvement underscores that lung disease in HCL typically represents 1 manifestation of a broader systemic process. Together, these patterns provide a coherent mechanistic explanation for the extent of dissemination observed and reinforce the need for thorough systemic evaluation and a high index of suspicion for multiorgan involvement whenever mycobacterial infection is considered in patients with HCL.

Distinguishing HCL progression from infection remains a clinically challenging task. Fever and generalized lymphadenopathy are uncommon in untreated HCL and should instead raise suspicion for opportunistic infection, especially *Mycobacterium* spp. [4, 14]. Similarly, hepatic, splenic, skin, and marrow lesions should not be attributed to leukemia alone without considering infection [58]. In endemic regions, mycobacterial infections may even precede or unmask HCL, and the co-occurrence of mycobacterial disease with splenomegaly, lymphomonocytopenia, or autoinflammatory features (eg, Sweet's syndrome, vasculitis, arthralgias) should prompt evaluation for underlying HCL [58]. While cladribine and pentostatin have transformed HCL outcomes, both induce profound and durable CD4⁺ lymphopenia, compounding infection risk [59]. Splenectomy, performed in nearly half of the reviewed cases, may further impair clearance of disseminated pathogens [60]. Vigilant infection surveillance is therefore critical at all stages of the disease, including before treatment, during therapy, and in remission.

The predominance of NTMs, particularly *M. kansasii* and MAC, raises therapeutic challenges. Unlike MTBC, for which treatment guidelines are well established, NTM management is more heterogeneous, with species-specific regimens that often require prolonged multidrug therapy and may be limited by significant toxicity [61]. This is particularly problematic in HCL patients who often have marrow suppression, hepatic dysfunction, or are receiving immunosuppressive chemotherapy [58]. Future research into optimized treatment strategies for NTM in hematologic malignancies is urgently needed, including the role of newer antimycobacterial agents.

From a practical standpoint, the findings highlight the importance of vigilance for infection even before starting HCL-directed therapy. Baseline screening for mycobacterial infection, including chest imaging and targeted microbiologic evaluation in endemic settings, could be considered for selected patients with unexplained systemic symptoms. While the concept of pre-emptive therapy against mycobacterial infections is appealing, its practical implementation remains extremely challenging. Hence, heightened clinical vigilance, timely microbiological investigations, and context-specific empiric therapy remain the most pragmatic approaches in such patients. These measures, while not yet supported by formal guidelines, may mitigate some of the excess mortality observed in this population. Moving forward, systematic collection of prospective data through multicenter registries could address several knowledge gaps. These include the true incidence of mycobacterial disease in HCL, comparative outcomes between MTBC and NTM infections, the impact of HCL therapy on infection risk, and optimal antimicrobial regimens. Integration of molecular pathogen diagnostics with immunophenotypic monitoring of HCL may further clarify the interplay between host immunity and pathogen persistence. Finally, research into prophylactic or pre-emptive strategies, whether antimicrobial, immunologic, or a combination, represents an unmet need for reducing infection-related mortality in this highly susceptible group.

Our analysis is limited by reliance on case reports and small case series, which are subject to publication bias, incomplete reporting, and heterogeneity in diagnostic and therapeutic approaches. Many cases predate modern microbiologic methods and current therapeutic standards, restricting direct applicability to contemporary practice. Nonetheless, pooling these data provides valuable insight into patterns of susceptibility, clinical presentation, and outcomes that would otherwise remain anecdotal.

## CONCLUSION

Patients with HCL demonstrate a clear predisposition to disseminated mycobacterial infections, reflected in the high proportion of cases presenting with multiorgan involvement. This susceptibility is consistent with the characteristic immune deficits of HCL, particularly monocytopenia, which compromises the host's defense against intracellular pathogens. Clinically, when patients with HCL develop fever, lymphadenopathy, or otherwise unexplained systemic inflammation, mycobacterial infection should be considered early, as presentations are frequently disseminated and atypical. The diversity of species identified, encompassing both MTB and multiple NTM pathogens, reinforces the importance of timely, species-level microbiological diagnosis to guide therapy. Collectively, our findings underscore several key takeaways: clinicians should maintain heightened awareness for mycobacterial disease in HCL; diagnostic evaluation should be prompt and thorough when compatible symptoms arise; and future research is needed to define incidence, understand comparative risks with other indolent hematologic malignancies, and improve early recognition and management strategies in this vulnerable population.

## Supplementary Material

ofag063_Supplementary_Data

## Supplementary Data

Supplementary materials are available at *Open Forum Infectious Diseases* online. Consisting of data provided by the authors to benefit the reader, the posted materials are not copyedited and are the sole responsibility of the authors, so questions or comments should be addressed to the corresponding author.
