# Supplementary material for: Mycobacterial Infections in Patients With Hairy Cell Leukemia: A Systematic Review of Published Cases
Source: Open Forum Infect Dis. 2026 Feb 11;13(2):ofag063. doi: 10.1093/ofid/ofag063 (PMC12915412; doi:10.1093/ofid/ofag063)
Supplement: ofag063_Supplementary_Data [file ofag063_supplementary_data.docx]

**Supplementary Materials Legend**

Supplementary File 1. PRISMA Checklist: Completed PRISMA 2020 checklist outlining adherence to systematic review reporting standards.

Supplementary Table 1. JBI Critical Appraisal of Included Case Reports.

Supplementary Table 2. Comparison of Clinical Profile and Outcomes Between Patients Who Developed Mycobacterial Infection Before vs After HCL Treatment.

Supplementary Table 3. Comparison of Clinical Features in Disseminated vs Non-Disseminated Mycobacterial Infection in HCL.

Supplementary Table 4. Comparison of MTBC vs NTM: Dissemination and Organ Involvement.

Supplementary Table 5. Organ Involvement Among Disseminated Cases: MTBC vs NTM.

Supplementary Table 6. Comparison of MTBC vs NTM Among Pulmonary Mycobacterial Cases.

**Supplementary Table 1.** JBI Critical Appraisal of Included Case Reports

| Sn | Case | **Demography** | **History** | **Presentation** | **Diagnosis** | **Treatment** | **Follow-up** | **Adverse event** |
| --- | --- | --- | --- | --- | --- | --- | --- | --- |
| 1 | Cellini | Yes | Yes | Yes | Yes | Yes | Yes | N/A |
| 2 | Castor | Yes | Yes | Yes | Yes | Yes | Yes | N/A |
| 3 | Broady | Yes | No | Yes | Yes | Yes | Yes | N/A |
| 4 | Haefliger | Yes | No | Yes | Yes | Yes | Yes | N/A |
| 5 | Dugdale | Yes | Yes | Yes | Yes | Yes | Yes | N/A |
| 6 | Bain | Yes | Yes | Yes | Yes | Yes | Yes | N/A |
| 7 | De Kruijf | Yes | Yes | Yes | Yes | Yes | Yes | N/A |
| 8 | Hulin | Yes | Yes | Yes | Yes | Yes | Yes | N/A |
| 9 | Trizna | Yes | No | Yes | Yes | Yes | Yes | N/A |
| 10 | Fleisher | Yes | No | Yes | Yes | Yes | Yes | N/A |
| 11 | Geller | Yes | Yes | Yes | Yes | Yes | Yes | N/A |
| 12 | Thaker | Yes | No | Yes | Yes | Yes | Yes | N/A |
| 13 | Thaker | Yes | No | Yes | Yes | Yes | Yes | N/A |
| 14 | Green | Yes | Yes | Yes | Yes | Yes | Yes | N/A |
| 15 | Valizadeh | Yes | Yes | Yes | Yes | Yes | Yes | N/A |
| 16 | Papadopoulos | Yes | Yes | Yes | No | Yes | Yes | N/A |
| 17 | Gogia | Yes | No | Yes | Yes | Yes | Yes | N/A |
| 18 | Weinstein | Yes | No | Yes | Yes | Yes | Yes | N/A |
| 19 | Weinstein | Yes | No | Yes | Yes | Yes | Yes | N/A |
| 20 | Manes | Yes | No | Yes | Yes | Yes | Yes | N/A |
| 21 | Raanani | Yes | No | Yes | Yes | Yes | Yes | N/A |
| 22 | Dave | Yes | No | Yes | Yes | Yes | Yes | N/A |
| 23 | Bennett | Yes | No | Yes | Yes | Yes | Yes | N/A |
| 24 | Bennett | Yes | No | Yes | Yes | Yes | Yes | N/A |
| 25 | Bennett | Yes | No | Yes | Yes | Yes | Yes | N/A |
| 26 | Bennett | Yes | No | Yes | Yes | Yes | Yes | N/A |
| 27 | Bennett | Yes | No | Yes | Yes | Yes | Yes | N/A |
| 28 | Bennett | Yes | No | Yes | Yes | Yes | Yes | N/A |
| 29 | Bennett | Yes | No | Yes | Yes | Yes | Yes | N/A |
| 30 | Bennett | Yes | No | No | No | Yes | Yes | N/A |
| 31 | Bennett | Yes | No | Yes | Yes | Yes | Yes | N/A |
| 32 | Rice | Yes | No | Yes | Yes | Yes | Yes | N/A |
| 33 | Rice | Yes | Yes | Yes | Yes | Yes | Yes | N/A |
| 34 | Weinstein | Yes | No | Yes | Yes | Yes | Yes | N/A |
| 35 | Weinstein | Yes | No | Yes | Yes | Yes | Yes | N/A |
| 36 | Mead | Yes | Yes | Yes | Yes | Yes | Yes | N/A |
| 37 | Maziarz | Yes | No | Yes | Yes | Yes | Yes | N/A |
| 38 | Hendrick | Yes | No | Yes | Yes | Yes | Yes | N/A |
| 39 | Maurice | Yes | Yes | Yes | Yes | Yes | Yes | N/A |
| 40 | Mummler | Yes | No | Yes | Yes | Yes | Yes | N/A |
| 41 | Fonseca | Yes | No | Yes | Yes | Yes | Yes | N/A |
| 42 | Ramasamy | Yes | Yes | Yes | No | Yes | Yes | N/A |
| 43 | Girardi | Yes | Yes | Yes | Yes | Yes | Yes | N/A |
| 44 | Filho | Yes | Yes | Yes | No | Yes | Yes | N/A |
| 45 | Kramers | Yes | Yes | Yes | Yes | Yes | Yes | N/A |
| 46 | Nielsen | Yes | No | Yes | Yes | Yes | Yes | N/A |
| 47 | Arslan | Yes | No | Yes | Yes | Yes | Yes | N/A |
| 48 | Stanton | Yes | No | Yes | Yes | Yes | Yes | N/A |

Each case report was assessed using the Joanna Briggs Institute (JBI) checklist for case reports. The table records whether the essential elements were adequately described: *Demography* (patient age/sex/background), *History* (timeline and prior conditions), *Presentation* (clinical features at presentation), *Diagnosis* (tests and results), *Treatment* (interventions provided), *Follow-up* (post-intervention outcomes), and *Adverse events* (harms or unexpected outcomes). Responses are indicated as “Yes” when adequately reported, “No” when absent, and “N/A” where not applicable.

**Supplementary Table 2. Comparison of clinical profile and outcomes between patients who developed TB before vs after HCL treatment**

| **Variable** | **Before HCL treatment (n = 30)** | **After HCL treatment (n = 18)** | **Total (N)** | **p-value** |
| --- | --- | --- | --- | --- |
| Fever | 28 (93.3%) | 15 (83.3%) | 48 | 0.272 |
| Sex (Male) | 24 (80.0%) | 15 (83.3%) | 48 | 0.775 |
| Monocytopenia | 13/14 (92.9%) | 9/9 (100%) | 23 | 0.412 |
| Lymphopenia | 9/13 (69.2%) | 6/9 (66.7%) | 22 | 0.899 |
| Disseminated TB* | 23/30 (76.7%) | 11/18 (61.1%) | 47 | 0.251 |
| Skin TB | 4 (13.3%) | 3 (16.7%) | 48 | 0.751 |
| CNS TB | 1 (3.3%) | 3 (16.7%) | 48 | 0.106 |
| Lymph Node TB | 25 (83.3%) | 4 (22.2%) | 48 | **<0.001** |
| Pulmonary TB | 17 (56.7%) | 10 (55.6%) | 48 | 0.940 |
| Pleural TB | 2 (6.7%) | 4 (22.2%) | 48 | 0.115 |
| Liver TB | 10 (33.3%) | 3 (16.7%) | 48 | 0.208 |
| Spleen TB | 9 (30.0%) | 3 (16.7%) | 48 | 0.302 |
| Bone marrow TB | 6 (20.0%) | 3 (16.7%) | 48 | 0.775 |
| Blood culture positive | 8/21 (38.1%) | 4/12 (33.3%) | 33 | 0.784 |
| Mortality | 13 (43.3%) | 6 (33.3%) | 48 | 0.493 |

**Legend & Footnotes:**
*Disseminated TB was defined as infection involving ≥2 non-contiguous organ systems or isolation of Mycobacterium spp. from a sterile site (blood, bone marrow, liver, or spleen).
Denominators differ for monocytopenia, lymphopenia, and blood culture due to missing data.*

**Supplementary Table 3. Comparison of clinical profile and outcomes in disseminated vs non-disseminated mycobacterial infection in HCL**

| **Variable** | **No Dissemination (n = 14)** | **Disseminated (n = 34)** | **Total (N)** | **p-value** |
| --- | --- | --- | --- | --- |
| Fever | 11 (78.6%) | 32 (94.1%) | 48 | 0.109 |
| Monocytopenia | 5/5 (100%) | 17/18 (94.4%) | 23 | 0.590 |
| Lymphopenia | 3/5 (60.0%) | 12/17 (70.6%) | 22 | 0.655 |
| TB after HCL therapy | 7 (50.0%) | 11 (32.4%) | 48 | 0.251 |
| Granulomas present | 8 (57.1%) | 22 (64.7%) | 48 | 0.623 |
| Mortality | 5 (35.7%) | 14 (41.2%) | 48 | 0.725 |
| Splenectomy performed | 5 (35.7%) | 17 (50.0%) | 48 | 0.367 |

**Legend & Footnotes:**
*Dissemination defined as per Supplementary Table 1 footnote.
Monocytopenia and lymphopenia values reflect only cases with available laboratory data.*

**Supplementary Table 4. Comparison of MTB vs NTM: Dissemination and Organ Involvement**

| **Variable** | **MTB = 1 (n = 12)** | **NTM = 2 (n = 32)** | **p-value** |
| --- | --- | --- | --- |
| Disseminated TB | 7 (58.3%) | 24 (75.0%) | 0.281 |
| TB skin involvement | 0 (0%) | 5 (15.6%) | 0.146 |
| CNS TB | **4 (33.3%)** | **0 (0%)** | **0.001** |
| Lymph node TB | 6 (50.0%) | 21 (65.6%) | 0.343 |
| Pulmonary TB | 6 (50.0%) | 19 (59.4%) | 0.576 |
| Pleural TB | 3 (25.0%) | 3 (9.4%) | 0.179 |
| Liver TB | 2 (16.7%) | 10 (31.3%) | 0.333 |
| Spleen TB | 2 (16.7%) | 9 (28.1%) | 0.434 |
| TB blood culture positive (valid n = 29) | 4/10 (40.0%) | 8/19 (42.1%) | 0.913 |
| Bone marrow TB | 2 (16.7%) | 7 (21.9%) | 0.703 |

**Legend & Footnotes:**
*MTB (1) includes Mycobacterium tuberculosis complex; NTM (2) includes all non-tuberculous mycobacteria.
Blood culture denominators reflect cases where mycobacterial culture data were available.
CNS involvement was significantly higher in MTB compared with NTM.*

**Supplementary Table 5. Organ Involvement Among Disseminated Cases: MTB vs NTM**

| **Variable** | **MTB (n = 7)** | **NTM (n = 24)** | **p-value** |
| --- | --- | --- | --- |
| Skin involvement | 0 (0%) | 3 (12.5%) | 0.325 |
| CNS TB | **2 (28.6%)** | **0 (0%)** | **0.007** |
| Lymph node TB | 5 (71.4%) | 17 (70.8%) | 0.976 |
| Pulmonary TB | 6 (85.7%) | 16 (66.7%) | 0.329 |
| Pleural TB | 2 (28.6%) | 3 (12.5%) | 0.309 |
| Liver TB | 1 (14.3%) | 10 (41.7%) | 0.183 |
| Spleen TB | 2 (28.6%) | 9 (37.5%) | 0.664 |
| TB blood culture positive (valid n = 23) | 4/5 (80.0%) | 8/18 (44.4%) | 0.159 |
| Bone marrow TB | 2 (28.6%) | 7 (29.2%) | 0.976 |

**Legend & Footnotes:**
*Includes only disseminated cases as per predefined criteria.
CNS involvement remained significantly higher in MTB even when analysis was restricted to disseminated disease.*

**Supplementary Table 6. Comparison of MTB vs NTM Among Pulmonary Mycobacterial Cases (n = 25)**

| **Variable** | **MTB (n = 6)** | **NTM (n = 19)** | **p-value** |
| --- | --- | --- | --- |
| **Disseminated TB** | 6 (100%) | 16 (84.2%) | 0.299 |
| **Skin involvement** | 0 (0%) | 1 (5.3%) | 0.566 |
| **CNS TB** | 2 (33.3%) | 0 (0%) | **0.009** |
| **Lymph node TB** | 4 (66.7%) | 14 (73.7%) | 0.739 |
| **Pleural TB** | 2 (33.3%) | 2 (10.5%) | 0.184 |
| **Liver TB** | 0 (0%) | 7 (36.8%) | 0.080 |
| **Spleen TB** | 1 (16.7%) | 5 (26.3%) | 0.629 |
| **TB blood culture positive** (valid n = 17) | 3/4 (75.0%) | 5/13 (38.5%) | 0.200 |
| **Bone marrow TB** | 1 (16.7%) | 3 (15.8%) | 0.959 |
